# Detection of wound healing disorders after major amputations by measurements of the microcirculation: A prospective single-center study

**DOI:** 10.1177/20503121241263244

**Published:** 2024-07-24

**Authors:** Katharina Zetzmann, Nikolaos Papatheodorou, Eva Rühl, Shatlyk Yagshyyev, Briain Haney, Oxana Moosmann, Yi Li, Alexander Meyer, Ferdinand Knieling, Christian-Alexander Behrendt, Werner Lang, Ulrich Rother

**Affiliations:** 1 Department of Vascular Surgery, University Hospital Erlangen, Friedrich-Alexander-Universität Erlangen-Nürnberg, Erlangen, Germany; 2 Department of Vascular Surgery, Helios Klinikum Berlin-Buch, Berlin, Germany; 3 Medical School Berlin, Berlin, Germany; 4 Department of Pediatrics, University Hospital Erlangen, Friedrich-Alexander-Universität Erlangen-Nürnberg, Erlangen, Germany; 5 Department of Vascular and Endovascular Surgery, Asklepios Klinik Wandsbek, Asklepios Medical School, Hamburg, Germany

**Keywords:** Major limb amputation, fluorescence angiography, indocyanine green, wound healing, microperfusion

## Abstract

**Introduction:**

Although major amputations can often be avoided due to evolving methods of endovascular and surgical revascularizations techniques, in patients with chronic limb-threatening ischemia, it is still necessary in some cases. Aim of this study was the detection of wound healing disorders through intraoperative microcirculation measurements in major limb amputations.

**Materials and methods:**

In this single-center clinical study, patients with an indication for major amputation were enrolled prospectively. Cause of amputation, patients’ comorbidities including cardiovascular risk profile were assessed. Macrocirculation, as well as microcirculation were assessed. Microcirculation measurements were performed by fluorescence angiography with the administration of indocyanine green. A preoperative measurement was obtained at the amputation level, followed by three additional measurements of the amputation stump postoperatively. Wound healing was monitored and correlated with the microcirculatory findings, based on the perfusion parameters ingress and ingress rate, calculated in the indocyanine green fluorescence video sequences of the amputation stumps.

**Results:**

Forty-five patients were enrolled, including 19 (42%) below-the-knee amputations and 26 (58%) above-the-knee amputations. When considering the need for revision, a change in the microperfusion parameters was observed postoperatively. The mean value for ingress was significantly lower directly postoperatively in stumps requiring revisions (5 ± 0 A.U. versus 40.5 ± 42.5 A.U., *p* < 0.001). The mean value of ingress rate behaved similarly (0.15 ± 0.07 A.U./s versus 2.8 ± 5.0 A.U./s, *p* = 0.005). The evaluation of indocyanine green measurements when wound healing disorders occurred also showed nonsignificant differences in the mean values.

**Conclusion:**

Fluorescence angiography after major lower limb amputations appears to be an option of depicting microperfusion. Especially, the early postoperative detection of reduced perfusion can indicate a subsequent need for revision. Therefore, this method could possibly serve as a tool for intraoperative quality control after major limb amputation.

## Introduction

Chronic limb-threatening ischemia (CLTI) is characterized as a clinical syndrome where peripheral artery disease (PAD) coexists with either rest pain, tissue necrosis, or lower limb ulceration lasting for more than 2 weeks according to recent ESVS guidelines.^1,2^

The prevalence of CLTI in the United States is estimated to be around 2 million, with an expected increase in the future. Hospitalization is frequent for CLTI patients, with up to 60% being readmitted within 6 months.^3^ For numerous years, the incidence of CLTI was approximated at 500 to 1000 new cases per million individuals in Western nations.^1^

In 2015, approximately 504,000 individuals in the United States were reported to be living with a major amputation resulting from PAD. Projections suggest this number to at least double by the year 2050.^1^ Beyond the risk of limb loss, CLTI patients also face a reduced life expectancy, often surpassing 50% mortality within 5 years.^3^

The SVS Threatened Limb Classification System (wound ischemia and foot infection (WIfI)) represents a notable advancement as a diagnostic tool, exhibiting a meaningful correlation with the probabilities of both limb salvage and wound healing subsequent to revascularization procedures.^1^ Given the intricate nature of addressing CLTI from an interdisciplinary standpoint, in conjunction with the prevalence of chronic wounds and the incorporation of WIfI, the imperative for diagnosing macro- and microcirculatory conditions in patients afflicted with CLTI is underscored.^1,3,4^ The diagnosis of CLTI is facilitated through noninvasive procedures, including the determination of the Ankle Brachial Index by abnormal values (<0.9), alongside assessments such as toe systolic pressure measurements or Doppler waveforms. Furthermore, microcirculatory assessments utilize approaches such as transcutaneous oxygen partial pressure (tcpO_2_), oxygen to see measurements, and Indocyanine green (ICG) fluorescence angiography.^1,3^ Noteworthy is the utilization of these modalities for discerning microcirculatory parameters. Multiple clinical investigations have substantiated a discernible correlation between measurements of macro- and microcirculation.^5–9^ There is consensus that in patients with PAD, the impact of comorbidities such as diabetes mellitus (DM), smoking, or chronic kidney disease (CKD) initially induces changes in microcirculation.^10^

Among the different methods of microcirculatory assessment, fluorescence angiography is a relatively new, noninvasive approach that can be used to detect disorders in tissue microcirculation in patients with CLTI or to predict wound healing disorders (WHD) after major amputations.^4,9^ The advantages of this method include high contrast, high sensitivity, being a reliable scientific tool, and ease of use.^11^

Fluorescence angiography is performed using the fluorescent dye ICG.^7,12–14^

Since ICG fluorescence angiography has attracted more and more interest in vascular medicine in recent years, this technique was used in this study for major amputations. The aim of the study was to use fluorescence angiography to detect WHD and thus make amputation surgery safer.

## Materials and methods

### Patients

A prospective single-center analysis was conducted consisting of 45 patients (33 men, 12 women; median age 73 years) who presented with peripheral arterial disease, diabetic foot syndrome, and critical limb-threatening ischemia and underwent major amputation. Study participants were enrolled between October 1, 2018 and January 1, 2023. In the absence of exclusion criteria, such as allergic diathesis or iodine allergy, patients were prospectively enrolled in the study. The study was conducted in congruence with the Declaration of Helsinki and the Declaration of Istanbul and was approved by the ethics committees of the University of Erlangen-Nürnberg (410_18B); all patients gave their written informed consent. The study adhered to the Standards for Reporting of Diagnostic Accuracy (STARD) guidelines.^15^

### Study design and procedure

#### SPY measurements

The measurement of microcirculation was conducted through fluorescence angiography using the SPY Elite^®^, Model LC 3000 (SPY Elite; Stryker^®^, Waterdown, Canada)^16^; ICG (VERDYE^®^; Diagnostic Green GmbH, Aschheim Dornach, Germany) was employed as the fluorescent dye.^17^ The microcirculation was tested in order to predict the tissue viability (microperfusion). ICG is a water-soluble tricarbocyanine dye that binds specifically to plasma proteins and globulins. As a result, this characteristic confines it to the intravascular space. The liver is the exclusive route for ICG elimination from the bloodstream, and due to the absence of entero-hepatic circulation, its half-life is approximately 3 min. With an absorption spectrum spanning from 600 to 900 nm, ICG can be activated by a near-infrared laser with a wavelength of 780 nm. This activation allows for tissue penetration of approximately 3 mm. Consequently, when ICG is localized in the subdermal microvasculature, it serves as an indicator of tissue perfusion. The unique optical properties of ICG, activated by near-infrared light, enable precise assessment of microvascular circulation in superficial tissues.^18^ ICG quickly diffuses, has a short half-life, and undergoes hepatic clearance as described above^19^; hence, it can be used in patients with CKD.^20^ Adverse drug events are minimal and scarce.^21,22^ Patients with history of allergic reactions to iodine or iodine-containing contrast agents should avoid this examination, to prevent hypersensitivity reactions.^6,17,19,23^ In this study, the ICG agent was used in the form of a dry powder.^17^ Each vial contained 25 mg of ICG. To prepare the injection solution, each vial was diluted with 10 mL of sterile sodium chloride solution 0.9% (Aqua). The injection solution was then used immediately. For each measurement, a standardized dose of 0.1 mg/kg body weight ICG was utilized. This dose aligns with the recommended single-dose for microcirculation diagnostics.^17,24^ The integrated laser sensor is programmed for a standardized distance of 30 cm between the limb and the imaging head.^24^ The measured limb should be positioned at least this far away. The two illuminating laser points connect to form a point on the wound, ensuring this distance. Intraoperatively, the predetermined amputation level was marked by the surgeon using locally applied ICG. Subsequently, ICG fluorescence angiography was performed. The SPY Elite’s near-infrared light camera was positioned vertically above the amputation level, and the operating room was darkened. Under continuous medical monitoring of the patients vital parameters, intravenous injection of ICG was administered. Illumination of the amputation level with a near-infrared light camera (800–810 nm) induced fluorescence in the ICG. Tissue perfusion was visualized through the integrated camera and displayed on the SPY Elite screen. Recording automatically stopped following the completion of the measurement. Subsequently, the AVI file in the SPY Elite was saved in the respective patient folder. After completing the amputation and closing the skin, the second intraoperative measurement was performed to visualize the perfusion of the wound.

**Figure 1.**
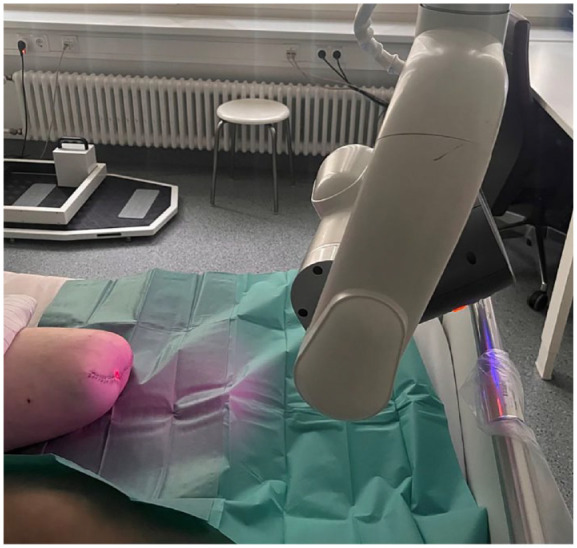
Measurement with SPY Elite.

The following measurements on the 5th and 10th postoperative day followed the same standardized study protocol (Figure 1):

1. Patient positioned in supine position.
2. Resting period of minimum 10 min.
3. SPY Elite near-infrared light camera positioned frontally to the amputation stump.
4. Input of patient data on the SPY Elite screen.
5. Preparation of ICG injection solution, covering the patient’s surroundings, and preparing all materials for ICG application.
6. Darkening of the patient’s room.
7. Intravenous ICG injection, followed immediately by intravenous NaCl infusion (20 mL).
8. Standardized recording after 136 s.
9. Manual saving of the AVI file in the patient folder of the SPY Elite.
10. Patient returned to their original position.

#### Analysis of fluorescence angiography video sequences

SPY Elite Analysis Software (SPY^®^-Q, NOVADAQ, Canada) ICG fluorescence measurements were saved as video sequences in AVI format and standardized evaluated using the integrated software. This software works on basis of a gray scale of 256 different shades, enabling analysis of fluorescence intensity. First, the “Fixed Baseline” setting was applied for the evaluation.^25,26^ A standardized intensity setting was used; in this study, set at two.

Subsequently, the video sequences of the patients were evaluated. Two video sequences per patient were loaded simultaneously into the evaluation window. Then, for each sequence to be evaluated, the entire area of the amputation level or wound edge was visually estimated, and then manually, the size of the rectangular region (ROI = “region of interest”) for the evaluation was determined. The size of the ROI for medial, central, and lateral evaluation was approximately one-third of the entire measurement area and remained the same after being set once per sequence.

These video sequences were then loaded into the evaluation program for each patient:

• Intraoperative (pre-op): amputation level
• Intraoperative (post-op): wound edge
• 5th post-op day: wound edge
• 10th post-op day: wound edge

**Figure 2.**
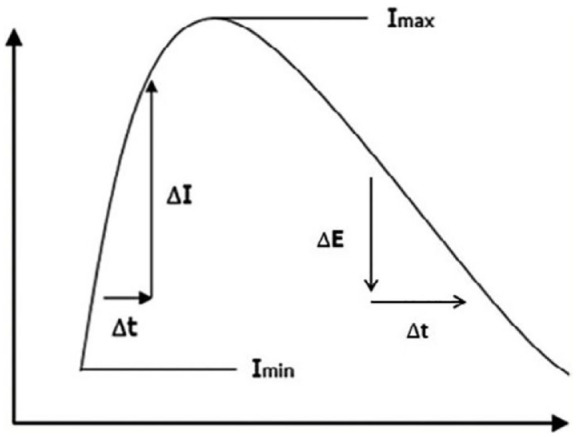
Evaluation of microcirculation parameters. *X*-axis: Time in seconds beginning after ICG injection; *Y*-axis: Fluorescence intensity in A.U.; InR: increase of fluorescence intensity per second (∆*I*/∆*t*); IN: maximum of fluorescence intensity subtracted from lowest fluorescence intensity (*I*_max_ − *I*_min_); EG: difference of the maximum of intensity and the end intensity (*I*_max_ − *I*_end_); EgR: decrease of the fluorescence intensity per second (∆*E*/∆*t*).^26^ ICG: indocyanine green.

A total of four measurement areas were evaluated for each video sequence: medial, central, lateral, and the entire wound length. For each measurement area, a graph was created, and two perfusion parameters were determined: ingress (IN) and ingress rate (InR)^3^; these serve to assess microcirculation and quantify ICG fluorescence angiography (Figure 2):

• IN (units): Difference between the maximum fluorescence intensity and the lowest fluorescence intensity.
• InR (units/s): Rate of increase in fluorescence intensity per second.

The four measurement areas were additionally evaluated in the photo documentation to ensure standardized com-parability

### Statistical analysis

All statistical calculations were performed using IBM SPSS Statistics 29.0.1.0 (IBM, Germany). Quantitative variables are presented as mean values together with minima and maxima and standard deviation. For qualitative factors, absolute and relative frequencies are given. The comparison of two independent groups was performed by a two-sample *t*-test, as appropriate. For all statistical tests, *p* < 0.05 was considered to show a statistically significant difference.

## Results

### Patient population

The prospective clinical study included 45 patients, of whom 33 were male and 12 were female. The patients were admitted to the hospital for various reasons, including CLTI (*n* = 40), Diabetic Foot syndrome (*n* = 3), and infected axillo-bifemoral bypass (*n* = 1). A total of 26 above-knee amputations and 19 below-knee amputations were performed. Fifteen patients (33%) were current smokers, while nine patients (20%) were former smokers. A total of 34 patients (76%) were taking anticoagulants. Patients’ comorbidities were described as follows: 89% (*N* = 40) of the patients had arterial hypertension, 51% (*N* = 23) had DM, 53% (*N* = 24) had dyslipidemia, and 16% had renal insufficiency, 2 (4%) of whom were undergoing dialysis. The remaining comorbidities of the patient population are summarized in Table 1.

**Table 1.** Patients’ demographic characteristics.

Age (mean, SD)	73, 11
Sex (male:female)	33:12
Above-the-knee amputations	26 (58%)
Below-the-knee amputations	19 (42%)
Comorbidities	
Art. hypertension	40 (89%)
Diabetes mellitus	23 (51%)
Dyslipidemia	24 (53%)
Currently smoking	15 (33%)
Former smoker	9 (20%)
Renal insufficiency under dialysis	2 (4%)
Cause of amputation	
Infected axillobifemoral bypass	1
CLTI	40
Diabetic foot syndrome	3
Acute extremity ischemia	3
Anticoagulation therapy	34 (76%)

### Evaluation of the overall perfusion over time

The perfusion parameters were investigated over time. The perfusion parameter Ingress Total (IN Total: Mean Ingress-measurement of the entire wound length) is higher preoperative than postoperative and increases in the postoperative course. On the 5th postoperative day, IN Total is higher than preoperative, and over the course of time, it is highest on the 10th postoperative day (Table 2). The preoperative perfusion parameter Ingress Rate Total (InR Total: Mean IngressRate of the entire wound length) is higher than postoperative and increases over the postoperative course. On the fifth postoperative day, IN Total is higher than preoperatively. On the 10th postoperative day, IN Total is the highest (Table 3). For the two perfusion parameters IN Total and InR Total, there is a significant reduction in perfusion (*p* < 0.001) from preoperative to postoperative measurement, as well as a significant perfusion increase (*p* < 0.001) from the 5th to the 10th postoperative day (Table 2). The absence of measurement values is justified by premature discharge of a patient to a nursing home (*n* = 1), a re-resection (*n* = 2), mortality (*n* = 1), and the defect of the fluorescence device (*n* = 1).

**Table 2.** Ingress of the entire wound and InR of the entire wound at the different time points of investigation.

IN (units)	Pre-op	Post-op	5th Post-op	10th Post-op
*n*	44	43	42	40
Mean	72.02	39.17	84.55	92.64
Min	16.00	5.00	14.00	21.00
Max	209.00	222.00	206.00	245.00
InR (units/s)	Pre-op	Post-op	5th Post-op	10th Post-op
*n*	44	43	42	40
Mean	3.80	2.63	5.05	6.60
Min	0.30	0.10	0.20	0.40
Max	33.70	22.50	35.80	44.10

InR: ingress rate.

**Table 3.** Ingress of the different wound areas (medial, central, lateral) at the different time points of investigation.

IN (units)	Pre-op	Post-op	5th Post-op	10th Post-op
IN wound medial				
*n*	44	43	42	40
Mean	56.06	30.18	71.28	82.84
Min	0.00	4.00	7.00	9.00
Max	181.00	120.00	236.00	240.00
IN wound central				
*n*	44	43	42	40
Mean	85.26	44.47	108.88	102.62
Min	0.00	0.00	16.00	14.00
Max	205.00	240.00	246.00	245.00
IN wound lateral				
*n*	44	43	42	40
Mean	55.71	37.84	75.62	86.15
Min	0.00	0.00	17.00	9.00
Max	225.00	155.00	211.00	237.00

IN: ingress.

### Evaluation of perfusion over time, depending on location

The perfusion parameter IN is higher medially, laterally, and centrally preoperatively than postoperatively and increases in the postoperative course. On the fifth postoperative day, IN is higher medially, laterally, and centrally than preoperatively. On the 10th postoperative day, IN is highest medially overall (Table 3). For the central and lateral locations, the behavior of IN is the same as for the medial location. When considering IN medially, centrally, and laterally over time, there is a significant reduction in perfusion (*p* < 0.001) from preoperative to postoperative and a significant perfusion increase (*p* < 0.001) from the 5th to the 10th postoperative day (Table 3).

The perfusion parameter InR is higher preoperatively and centrally than postoperatively (Table 4). On the fifth postoperative day, InR is higher than preoperatively both medially, laterally, and centrally. On the 10th postoperative day, InR is higher than preoperatively both medially and laterally. Centrally, InR continues to increase from the 5th to the 10th postoperative day and is highest on the 10th postoperative day.

**Table 4.** InR of the different wound areas (medial, central, lateral) at the different time points of investigation.

InR (units/s)	Pre-op	Post-op	5th Post-op	10th Post-op
InR wound central				
*n*	45	43	42	40
Mean	2.89	1.74	4.51	6.78
Min	0.00	0.00	0.40	0.20
Max	23.00	8.90	34.60	99.00
InR wound lateral				
*n*	45	43	42	40
Median	2.49	2.56	4.63	8.32
Min	0.00	0.00	0.50	0.30
Max	22.40	18.20	33.30	99.00
InR wound medial				
*n*	45	43	42	40
Mean	4.64	2.62	7.11	9.42
Min	0.00	0.10	0.40	0.40
Max	37.60	25.00	42.00	99.00

InR: ingress rate.

Over time, InR shows a significant perfusion reduction (*p* < 0.001) from pre- to postoperative medial and again a significant perfusion increase (*p* < 0.001) from the 5th to the 10th postoperative day both medially and laterally (Table 4). In the course of the measurements, a significant reduction in perfusion (*p* < 0.001) from preoperative to postoperative measurements is observed centrally for InR. Additionally, there is a significant increase in perfusion (*p* < 0.001) between the 5th and 10th postoperative day (Table 4).

### Evaluation of wound healing based on perfusion assessment

**Figure 3.**
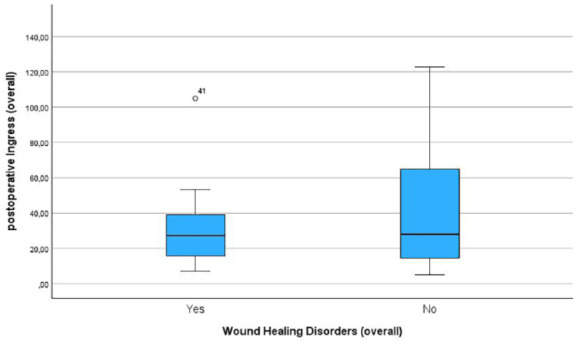
Boxplots showing the postoperative ingress in patients developing wound healing disorders and those with primary wound healing.

**Figure 4.**
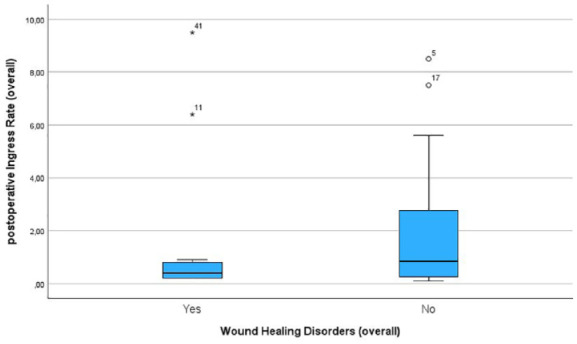
Boxplots showing the postoperative InR in patients developing wound healing disorders and those with primary wound healing.

In total, WHD were observed in 14 patients. Out of the 26 patients undergoing above-the-knee amputation, 6 developed WHD. Among the 19 patients who underwent below-the-knee amputation 8 developed WHD. The postoperative Ingress value was lower in patients with WHD compared to patients without WHD (33.5 ± 37.4 A.U. in patients with WHD, 43 ± 45.2 A.U. in patients without WHD) (Figure 3). The same trend was observed for the postoperative InR for those patients that developed WHD (1.8 ± 3 A.U./s in patients with WHD, 2.92 ± 5 A.U./s in patients without WHD) (Figure 4). However, these results were not statistically significant.

**Figure 5.**
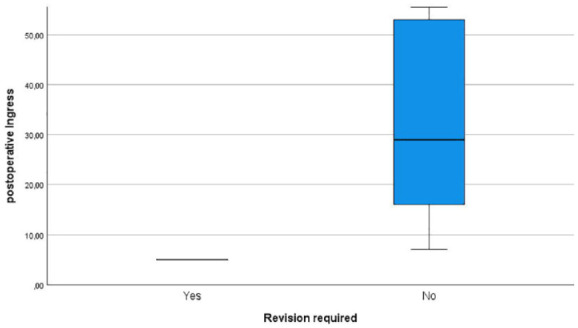
Boxplots showing the postoperative ingress in patients requiring wound revision and those with primary wound healing.

**Figure 6.**
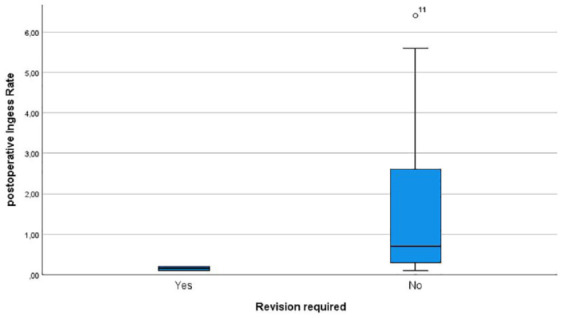
Boxplots showing the postoperative ingress rate in patients requiring wound revision and those with primary wound healing.

When considering the need for revision, a total of 2 patients required revision procedures due to WHD. In these patients, a significant microperfusion deficit was observed in the early postoperative ICG angiography (Figure 5). The mean value for IN was significantly lower directly postoperatively in stumps requiring revisions (5 ± 0 A.U. versus 40.5 ± 42.5 A.U., *p* < 0.001). The mean value of InR behaved similarly (0.15 ± 0.07 A.U./s versus 2.8 ± 5.0 A.U./s, *p* = 0.005) (Figure 6).

## Discussion

CLTI poses a challenge for vascular surgeons due to the high rates of morbidity and mortality. Although revascularization strategies improved in recent years, there will still remain a relevant percentage of patients receiving major amputations. It is therefore crucial that this last procedure in particular can be carried out safely with as few complications as possible. A decisive factor in achieving wound healing in this critically ill patient cohort is adequate wound perfusion. Therefore, the utilization of an effective intraoperative perfusion assessment concept could possibly be a way to reduce WHD.

In this study, ICG fluorescence angiography was therefore implemented in the amputation procedure. Therefore, we used ICG-based fluorescence angiography in this study. This is a relatively nontoxic and albumin-bound compound, which has extensive applications in various medical fields, particularly in hepatic, cardiac, and ophthalmologic studies. Its utilization in analyzing tissue perfusion and identifying sentinel lymph nodes in cancer staging is increasingly gaining importance. Since the 1950s, ICG has been employed in medical applications for quantitatively measuring hepatic and cardiac function. Following administration, ICG typically binds to plasma proteins intravascularly, undergoes hepatic uptake, and is subsequently excreted into bile. The hepatic clearance of ICG typically occurs within 10 to 20 min postadministration. The maximal clearance velocity of the ICG dye is 3.6 mg/kg/min, with a reduction observed in patients with liver pathologies such as cirrhosis.^27^ Current medical applications of ICG encompass cardiac vessel angiography, ophthalmology procedures (including macular hole surgery and ophthalmic angiography), real-time imaging for abdominal surgery (detecting colorectal anastomotic leaks, enhancing lymph node harvest in uterine cancer oncologic staging, evaluating abdominal wall perfusion, and preventing skin necrosis in mastectomies), intraoperative assessment of flap perfusion in reconstructive procedures,^28–33^ including the transplantation of free skin flaps,^34^ and tissue perfusion measurements in amputation stumps in the field of vascular surgery.^18,35^ Additionally, ICG plays a pivotal role in translational medicine, particularly in kidney transplantation, where microperfusion is being intraoperatively evaluated.^36^ Its applications also extends to neurosurgery, aiding in the detection of arteriovenous malformations. Moreover, it is being used in identifying chyle leaks, preventing bile leaks posthepatectomy, and highlighting the biliary system during cholecystectomy and Whipple procedures.^19,31^ These multifaceted applications underscore the significance of ICG as a versatile and valuable tool in diverse medical settings.^27^ The application of ICG fluorescence angiography after major limb amputation is relatively new and has only been described once before in a pilot study setting.^14^ Surgical certainty of perfusion situations is of high necessity, especially in critical ill patients like the CLTI cohort. Therefore, ICG fluorescence angiography can support intraoperative decision-making, as perfusion can be directly visualized for the surgeon. As shown in this study, malperfusion of wounds can be visualized, and quantitatively assessed by this method. Although the study cohort is too small to characterize the results as convulsive, there are hints that those amputated limbs requiring surgical revision in the later turn, could already be detected intraoperatively. This would enable surgeons to react to the findings during the operation, for example, by proximalizing the amputation height.

Similar attempts have been already made using different methods of perfusion assessment. The best known of these is the use of tcpO_2_ for the detection or prediction of wound healing. As this has been in use for decades, there are numerous studies on this, including a recent review. However, the conclusion from this is merely that wound healing is likely at a perfusion pressure of >40 mmHg. Absolute cut-off values for the production of wound healing do not exist for this either.^37^ The disadvantage of using tcpO_2_ intraoperatively is certainly the long calibration period and the fact that only punctual measurement is possible with the attached probes. In contrast, ICG fluorescein angiography has the advantage of visualization and immediate intraoperative information acquisition.

Avoiding WHD and surgical site infections as most common postoperative complications is essential in major limb amputation. This can potentially lead to a long-term reduction in postoperative revisions, decreased hospitalization duration, early transition to rehabilitation centers, cost reduction, improvement in patients’ quality of life, sepsis, and death, as recently published articles demonstrate.^38,39^

### Limitations

This study shows some limitations. The data reported here is from a single study center. This results in the limited number of patients. The number of cases in this study is not based on a case number calculation including a power analysis. Therefore, the results of this study should not be considered conclusive. Even though this represents the largest cohort reported to date, the limited number of patients excludes larger subgroup analyses. It is also not possible to perform a confounder analysis in this cohort. The near-infrared light spectrometry technique used here has limitations in terms of penetration depth into tissue. Therefore, it is only possible to make statements about the uppermost tissue layer, as the deep subcutaneous layers are not imaged.

Despite these limitations, intraoperative measurement of stump perfusion provides a means of quality control. The study protocol presented here could serve as a template for future larger-scale studies in order to generate conclusive results and comparable cut-off values.

## Conclusion

Intraoperative and postoperative fluorescence angiography after major lower limb amputations appears to be an effective method of depicting microperfusion. Especially, the early postoperative detection of reduced perfusion can indicate a subsequent need for revision. Therefore, this method might serve as a tool for intraoperative quality control after major limb amputation. However, a larger number of patients might help determine the significance of this aspect.
